# Online Design Aid for Evaluating Manure Pit Ventilation Systems to Reduce Entry Risk

**DOI:** 10.3389/fpubh.2016.00108

**Published:** 2016-05-26

**Authors:** Harvey B. Manbeck, Daniel W. Hofstetter, Dennis J. Murphy, Virendra M. Puri

**Affiliations:** ^1^Agricultural and Biological Engineering Department, Pennsylvania State University, University Park, PA, USA

**Keywords:** computer simulation, contaminant gas evacuation, manure pit ventilation, agricultural safety, oxygen replenishment

## Abstract

On-farm manure storage pits contain both toxic and asphyxiating gases such as hydrogen sulfide, carbon dioxide, methane, and ammonia. Farmers and service personnel occasionally need to enter these pits to conduct repair and maintenance tasks. One intervention to reduce the toxic and asphyxiating gas exposure risk to farm workers when entering manure pits is manure pit ventilation. This article describes an online computational fluid dynamics-based design aid for evaluating the effectiveness of manure pit ventilation systems to reduce the concentrations of toxic and asphyxiating gases in the manure pits. This design aid, developed by a team of agricultural engineering and agricultural safety specialists at Pennsylvania State University, represents the culmination of more than a decade of research and technology development effort. The article includes a summary of the research efforts leading to the online design aid development and describes protocols for using the online design aid, including procedures for data input and for accessing design aid results. Design aid results include gas concentration decay and oxygen replenishment curves inside the manure pit and inside the barns above the manure pits, as well as animated motion pictures of individual gas concentration decay and oxygen replenishment in selected horizontal and vertical cut plots in the manure pits and barns. These results allow the user to assess (1) how long one needs to ventilate the pits to remove toxic and asphyxiating gases from the pit and barn, (2) from which portions of the barn and pit these gases are most and least readily evacuated, and (3) whether or not animals and personnel need to be removed from portions of the barn above the manure pit being ventilated.

## Introduction

On-farm manure storage pits contain both toxic and asphyxiating gases. The primary gases of concern are hydrogen sulfide, ammonia, carbon dioxide, and methane. Occasionally, farm workers must enter the manure storage pits for maintenance and repair. Most farms do not have self-contained breathing devices; many do not have toxic and asphyxiating gas detection devices. Consequently, farm workers often enter the manure pits unprotected, lose consciousness, and die. Tragically, such incidents often result in multiple deaths as an observing worker tries to assist the one originally overcome by the toxic and asphyxiating gases. Beaver and Field (1) summarized documented fatalities in livestock manure storage and handling facilities from 1975 to 2004. One result from this analysis of 77 fatalities cases showed an increasing trend in the death rate: 1.6 per year from 1975 through 1984, 2.7 per year from 1985 through 1994, and 3.5 per year from 1995 through 2004.

**Figure 1:**
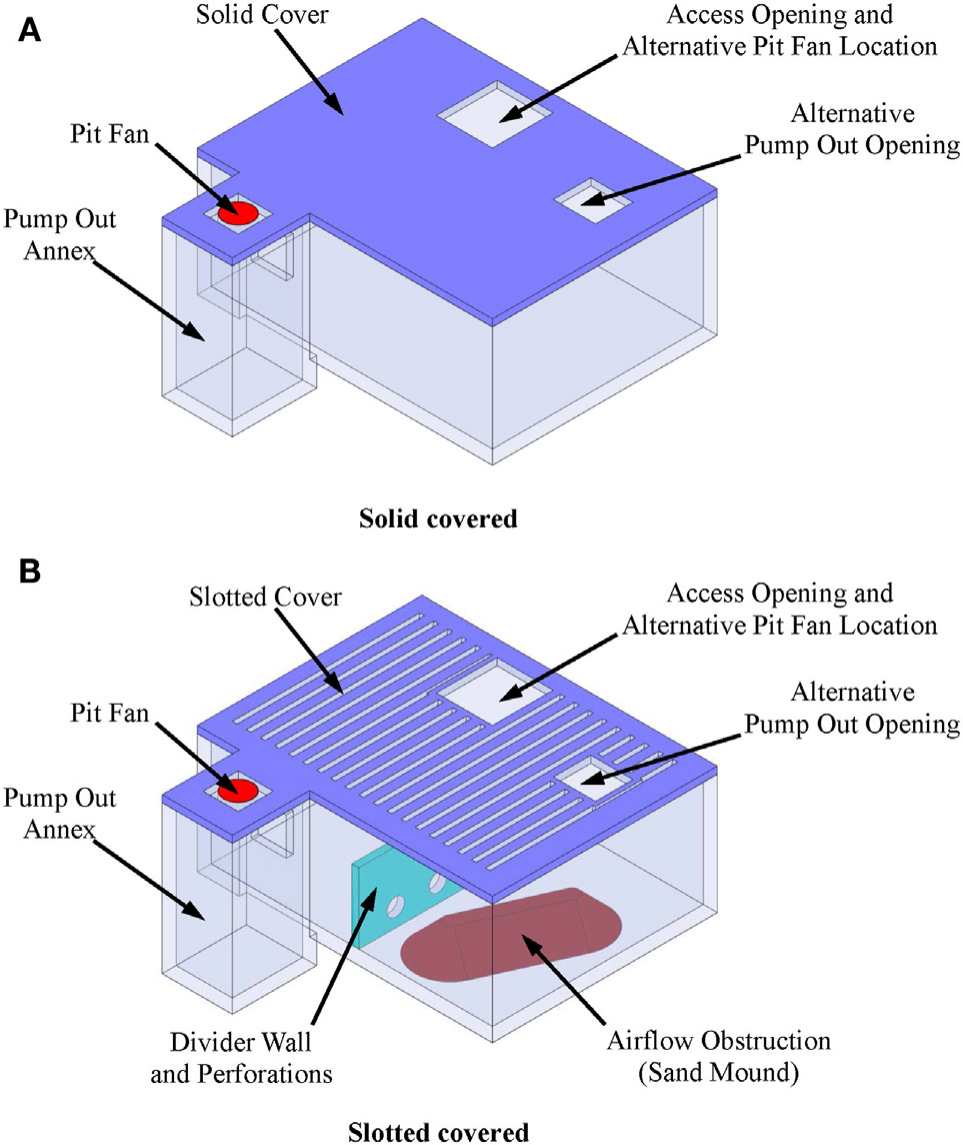
**Definition sketch of stand-alone manure pits with (A) solid cover and (B) slotted cover illustrating online tool additional features**.

**Figure 2:**
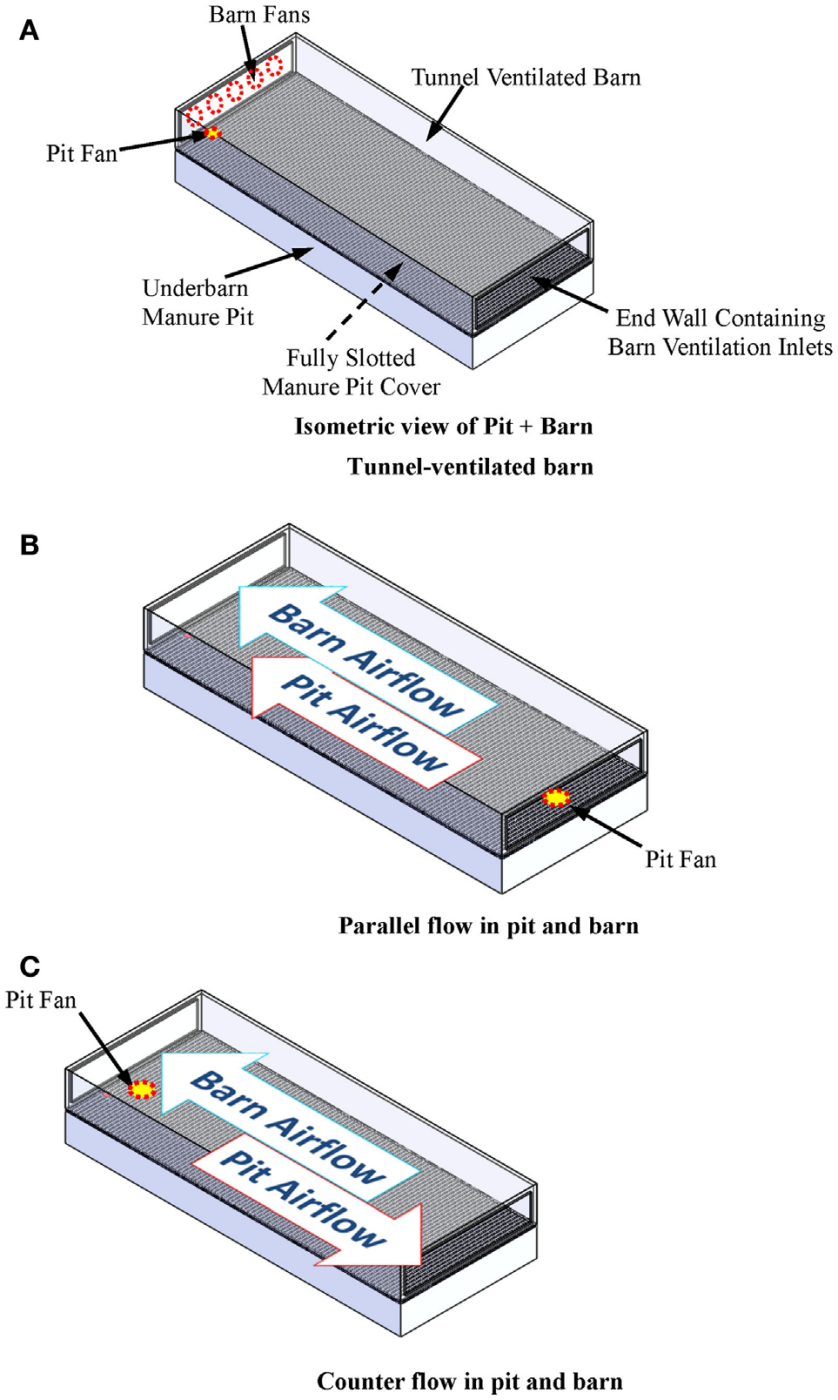
**(A)** Definition sketch of a slotted-covered manure pit beneath a tunnel-ventilated barn; **(B)** parallel flow in tunnel-ventilated pit + barn; and **(C)** counter flow in tunnel-ventilated pit + barn.

**Figure 3:**
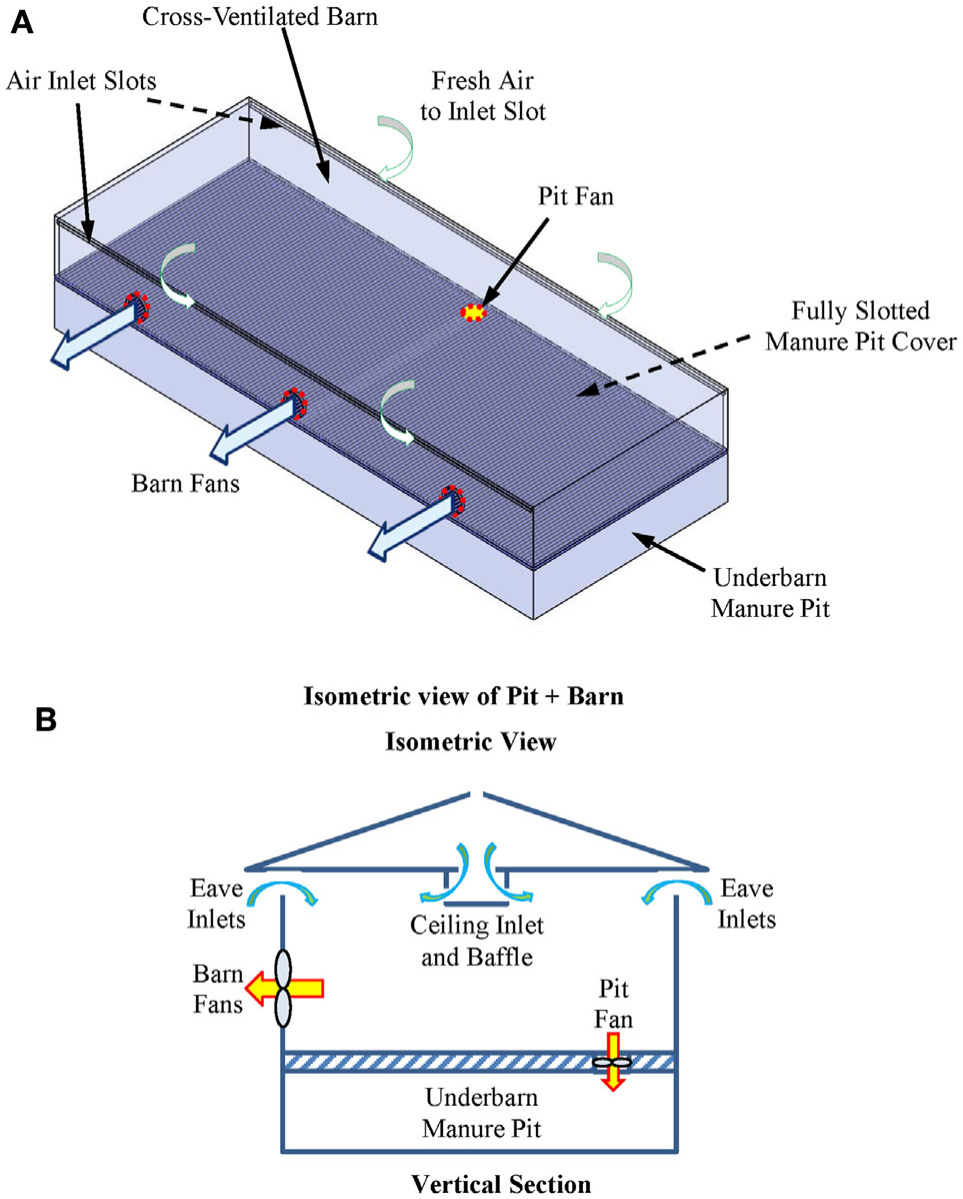
**Definition sketch of a slotted-covered manure pit beneath a cross-ventilated barn**. **(A)** Isometric view and **(B)** vertical section showing general airflow direction in barn.

**Figure 4:**
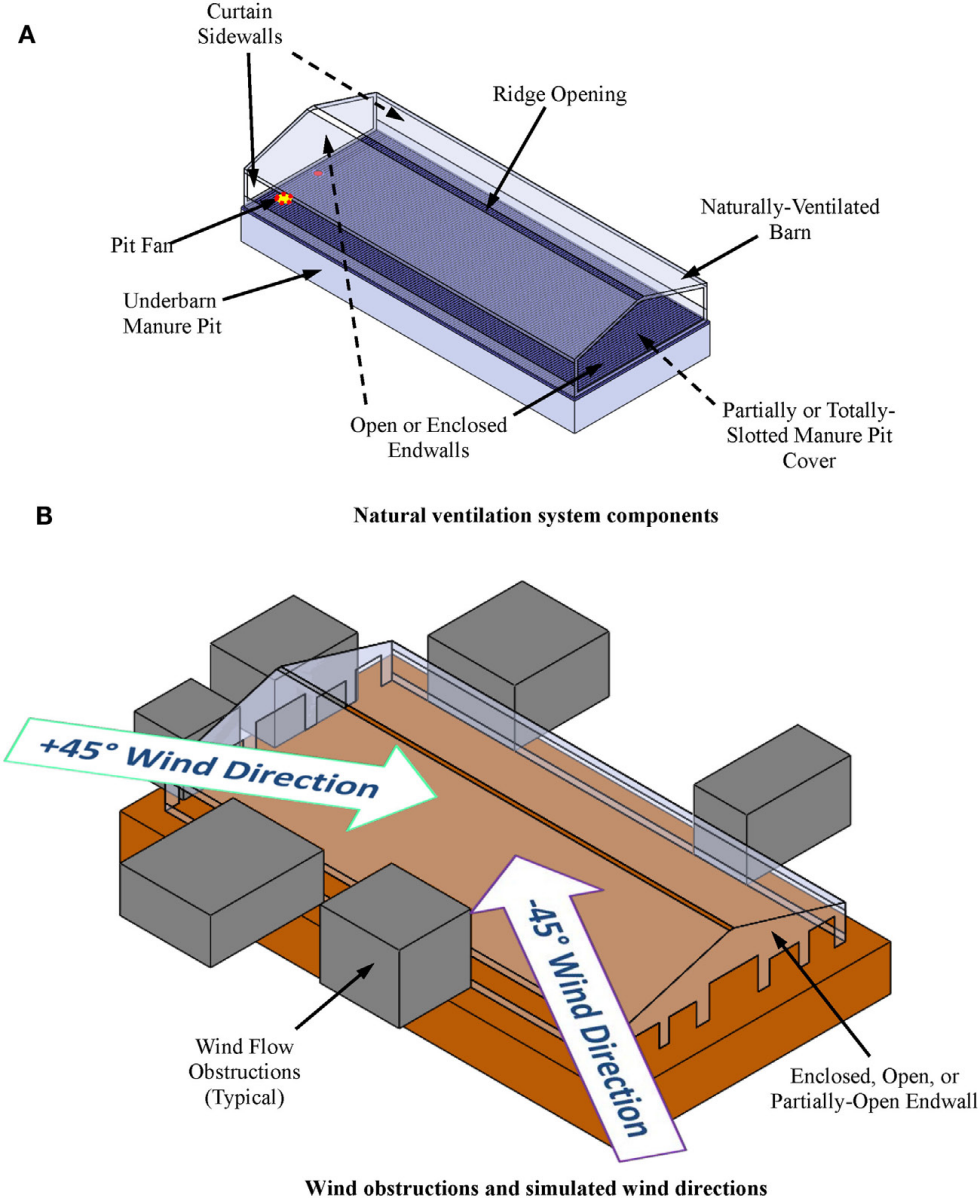
**Definition sketches for naturally ventilated barn above slotted-covered manure pit**. **(A)** Natural ventilation system components and **(B)** wind obstructions and wind directions simulated by online design aid.

One intervention to reduce the toxic and asphyxiating gas exposure risk to farm workers entering the manure pits is manure pit ventilation. The basic questions then become: (1) how much and for how long must the manure pit be ventilated to reduce entry risk? and (2) does the manure pit ventilation contaminate portions of the barn above manure pits during pit ventilation? This article describes a user-friendly, online computational fluid dynamics (CFD)-based design aid for evaluating the effectiveness of manure pit ventilation systems to reduce the concentrations of toxic and asphyxiating gases in manure pits. To the best of our knowledge, this is the first online CFD-based manure pit ventilation system design aid and analysis tool available to agricultural waste facilities planners, agricultural building design professionals, industrial hygienists, regulatory agencies, and emergency responders. The online design aid is organized into four modules, one each for solid-covered stand-alone manure pits (Figure 1), slotted-covered manure pits beneath tunnel-ventilated barns (Figure 2), slotted-covered manure pits beneath cross-ventilated barns (Figure 3), and slotted-covered manure pits beneath naturally ventilated barns (Figure 4).

The design aid is intended to be used primarily by waste management specialists, animal facilities designers, engineers, agricultural safety specialists, emergency rescue, and regulatory personnel who wish to assess the effectiveness of existing or alternative manure pit ventilation systems to remove contaminant gases and replenish oxygen prior to personnel entry. Typical applications of the design aid include (1) screening alternative ventilation system layouts to determine which is most effective for removing contaminant gases; (2) estimating required manure pit ventilation times to evacuate contaminant gases for a given ventilation system layout to concentrations suitable for human long-term occupancy (2, 3); (3) estimating required manure pit ventilation times to replenish oxygen levels to 20% by volume (3); and (4) determining which areas of barns above slotted-covered manure pits become contaminated to levels requiring evacuation of animals and personnel prior to and during manure pit ventilation.

## Equipment Requirements

The design aid resides on a Pennsylvania State University server. It consists of (1) a pre-processing package in which user input, such as basic building and ventilation system data, is transformed into a format suitable for the CFD simulation program; (2) the CFD software package SolidWorksFlowSimulation^®^ (SWFS^®^); (3) a preview module that allows a user to view a 3-D visualization of the input data; and (4) a post-processing module that retrieves and transforms the SWFS^®^ results into a user-friendly format. User minimum computer software requirements for inputting, accessing, and interpreting design aid results are (1) the latest version of Internet Explorer, Firefox, or Chrome; (2) Microsoft Excel^®^ 2010; and (3) the latest version of Adobe Reader.

## Background and Supporting Research

The supporting published research for development of the design aid was conducted and reported by a Pennsylvania State University research team in a series of five journal articles (4–8). These journal articles served as the basis for development and publication of a peer reviewed and American National Standards Institute (ANSI) approved engineering standard, ANSI/ASABE Standard S607, on ventilation of confined-space manure storages to reduce entry risk (9, 10). This is the first engineering standard to address specific ventilation strategies, including fan location, outlet location, air exchange (AC) rates, and ventilation times required to reduce contaminant gases in confined-space manure storages to below either Occupational Safety and Health Administration (OSHA)-defined personal exposure levels (PELs) or American Conference of Governmental Industrial Hygienists (ACGIH)-defined threshold limit values (TLVs) for hydrogen sulfide, carbon dioxide, and methane, and to replenish oxygen levels from 0% to ACGIH-defined TLVs for oxygen.

Occupational Safety and Health Administration has developed confined-space regulations documented in the 29 Code of Federal Regulations (CFR) Part 1910.146 (11). Manure storages are confined spaces, but Agriculture was exempted from OSHA’s 1910.146 standard when it first passed in 1993. Even so, the authors have used standard 1910.146 as a reference point for the atmospheric hazards associated with confined-space manure storages. These regulations require that the internal atmosphere within a confined space be tested for oxygen levels, flammable gases and vapors, and potential noxious contaminants prior to human entry. According to OSHA standards, an employee may not enter a confined space until forced-air ventilation has eliminated any existing hazardous atmosphere. Thus, it is imperative that confined spaces be properly ventilated prior to entry. The OSHA-defined PEL for hydrogen sulfide is 10 ppm; the ACGIH-defined TLV for hydrogen sulfide is 1 ppm (2, 3). The ACGIH-defined TLVs for methane and ammonia are 1,000 and 25 ppm, respectively (3). The OSHA-defined PEL for carbon dioxide is 5,000 ppm (2). The ACGIH-defined TLV for O_2_ in confined spaces prior to entry is 19.5% by volume up to an altitude of 1,525 m (3).

In experimental studies, hydrogen sulfide (H_2_S) was used as an indicator gas to investigate the effectiveness of forced-ventilation strategies for eliminating the toxic and oxygen-deficient atmospheres in the confined-space manure pits. Typical H_2_S concentration reduction curves during forced-air ventilation were identified in a rectangular manure tank. Based on the experimental tests conducted in the research, the most promising candidate ventilation strategies were identified for the studied rectangular confined-space manure tank with solid, totally slotted, and partially slotted covers. In addition, based on results of experimental tests, a field-based database was developed for the validation of CFD modeling protocols (4). As an important input parameter of the CFD modeling protocols, manure gas emissions were measured experimentally using the same rectangular tank. The influencing factors on gas emissions were identified as well (5).

The CFD modeling protocols to simulate H_2_S removal from fan-ventilated confined-space manure storages were developed and validated. The CFD model was used to conduct the simulations of evacuating H_2_S during forced ventilation for the best ventilation strategies identified in the work by Pesce et al. (4) for a typical rectangular on-farm manure tank with three cover types (i.e., solid, totally slotted, and partially slotted). Validation of the CFD modeling protocols was based on comparisons between simulated and measured H_2_S evacuation times. Simulated and measured evacuation times within the confined-space manure storage facilities evaluated agreed within 10% at all measuring locations except those immediately adjacent to the ventilation fan jet for all three cover types for both high (5 AC min^−1^) and low (3 AC min^−1^) AC rates. Corresponding evacuation times agreed to within 15% for all cover types and AC rates in the high-velocity gradient region of the ventilation fan jet. Having agreement to within 15% in these high-velocity gradient zones was justified because contaminant gas concentrations in these regions were evacuated rapidly to very low levels, and small differences in measuring locations would produce additional percentage differences in results. These results demonstrated that the CFD modeling protocols developed satisfactorily predict the gas concentration decay during forced ventilation in confined-space manure pits (6). The validated CFD modeling protocols were then applied to conduct simulations for identifying manure gas evacuation times and oxygen level recovery in the confined-space manure pits with different footprints. The factors (i.e., AC rate, manure gas emission rates, and gas initial concentration) influencing the gas evacuation time were identified (7, 8).

Design engineers and agricultural building planners often use one of the several computer-aided design (CAD) software packages, such as SWFS^®^ (12–14), for many design applications, especially for more complex tasks. Assessing the performance of ventilation systems for confined-space manure storages is a fairly complex engineering task for which a CAD software package with CFD capability is very useful. The SWFS^®^ software package includes a user-friendly CFD application suitable for simulating and designing ventilation systems for evacuating contaminant gases from and replenishing oxygen in confined-space manure storages. The SWFS^®^ software package was used in conjunction with the CFD simulation protocols developed by the Pennsylvania State University research team to develop the online design aid.

The suitability of the SWFS^®^ CFD software for the online tool development was verified by comparing SWFS^®^ simulated H_2_S gas decay vs. ventilation responses to those measured by Pesce et al. (4) and simulated using Phoenics as reported by Zhao et al. (5). These comparisons were made for H_2_S gas decay in a 2.74-m wide by 5.49-m long by 1.83-m deep solid covered manure tank at two ventilation rates (high – 5 AC/m and low – 1.0 AC/m). The best agreement between the Phoenics simulated and measured results were obtained with a CFD simulation time step size of 10 s. The Phoenics simulation results were validated by successfully matching the simulated and measured H_2_S decay vs. ventilation time response at 10 locations within the tank (5 upper level locations and 5 lower locations). Upper level location *R*^2^ values for simulated vs. measured decay curves were 0.94 and 0.85, respectively, for the high and low AC rates. Corresponding *R*^2^ values for the lower level grid locations were 0.88 and 0.93. The SWFS^®^ simulated H_2_S decay curves also were in excellent agreement with the measured decay data for the manure pit described by Pesce et al. (4). For the high AC rate, a 5-s time step provided the best match with the measured data for the upper grid (*R*^2^ = 0.94). A 7-s time step provided the best match with the measured data for the lower grid (*R*^2^ = 0.85). For the low AC rate, a 5-s time step provided the best match for both the upper (*R*^2^ = 0.81) and lower (*R*^2^ = 0.88) grid locations.

## Stepwise Procedures

### Underlying Assumptions and Justifications

The primary assumptions for development of the online tool are (1) the manure pit is ventilated with a positive pressure ventilation system; (2) there are no ventilation air distribution ducts inside the manure pit; (3) the barn above a slotted-covered manure pit is negative pressure ventilated at the design hot weather ventilation rate for a fully stocked animal facility prior to and during pit ventilation; (4) the manure pit is nearly empty (i.e., only residual manure of approximately 150 mm or less remains inside the pit); (5) the contaminant gas concentrations are initially uniformly throughout the manure pit domain; (6) initial oxygen levels inside the manure pit are 0% by volume; and (7) the barn atmosphere is free of contaminant gases, and the oxygen content is 20.9% by volume prior to manure pit ventilation.

Positive pressure pit ventilation is assumed because many manure pit configurations, especially those with slotted covers, are prone to short circuiting of ventilation air flow patterns. Positive pressure ventilation systems are less prone to short circuiting than are negative pressure ventilation systems. Ventilation air distribution ducts large enough to provide the manure pit AC capacity required for removal of contaminant gases to levels suitable for pit entry in a reasonable ventilation time frame are not practical. They significantly reduce manure pit capacity and are expensive to install. In addition, in existing facilities, installing a satisfactory temporary air distribution duct is not a reasonable option. Since installation of such distribution ducts is an essential component of satisfactory ventilation of short circuiting prone manure pits ventilated with negative pressure systems, the first underlying assumption is further justified. The third assumption is imposed to minimize the cross-contamination of the barn space above slotted-covered manure pits during positive pressure ventilation of the pit. Any properly designed ventilation system in the barn above a manure pit will have a ventilation system capacity able to achieve the design hot weather ventilation rate defined in ASABE EP270.5 (9) and MWPS (15) for a fully stocked animal facility. Assumption four is imposed for practical safety considerations. Assumption five is justified and conservative if initial gas concentration levels are measured at several locations inside the manure pit prior to pit ventilation and the maximum measured concentration used for the initial condition. Assumption six assures a conservative initial condition and required pit ventilation time before entry. Assumption seven is obtained by ventilating the barn above slotted-covered manure pits at the hot weather rate for approximately 2–5 min prior to manure pit ventilation.

Other design aid module-specific assumptions are imposed. These are identified in the more detailed presentations of the individual design aid modules.

### Accessing the Design Aid

The design aid is accessed at the website: https://ventdesign.agsafety.psu.edu. Users follow the prompts to register to use the online design tool and are notified by email that they can submit projects.

### Input Data Entry

The user is prompted to complete a general information form. This includes some demographic information about the user and the animal enterprise for the current project submission. Then, the user is prompted to enter project-specific data necessary to characterize the manure pit and barn geometry, the pit ventilation system, and the barn ventilation system. Data entry for the online design aid is in English units. This unit system was selected because English units are the preferred platform for the vast majority of anticipated design aid users. Only two soft conversions are required to convert all required inputs from SI to English units: (1) the size and location of geometric features from meters to feet and inches and (2) fan airflow capacity from cubic meters per second to cubic feet per minute.

#### Selection of the Simulation Module

The user selects the simulation module that best describes the project manure pit and barn. The four simulation modules are (1) stand-alone manure pits (Figure 1), (2) tunnel-ventilated barns above slotted floor covered manure pits (Figure 2), (3) cross-ventilated barns above slotted floor covered manure pits (Figure 3), and (4) naturally ventilated barns above slotted floor covered manure pits (Figure 4). Upon simulation module selection, the user is directly transferred to the specific data input pages for the project. Input protocols for stand-alone manure pits are first presented followed by protocols for barns above slotted-covered manure pits. Many input protocols are common across all modules; however, some are unique to a given simulation module.

#### Inputs for Stand-alone Manure Pits

**Figure 5:**
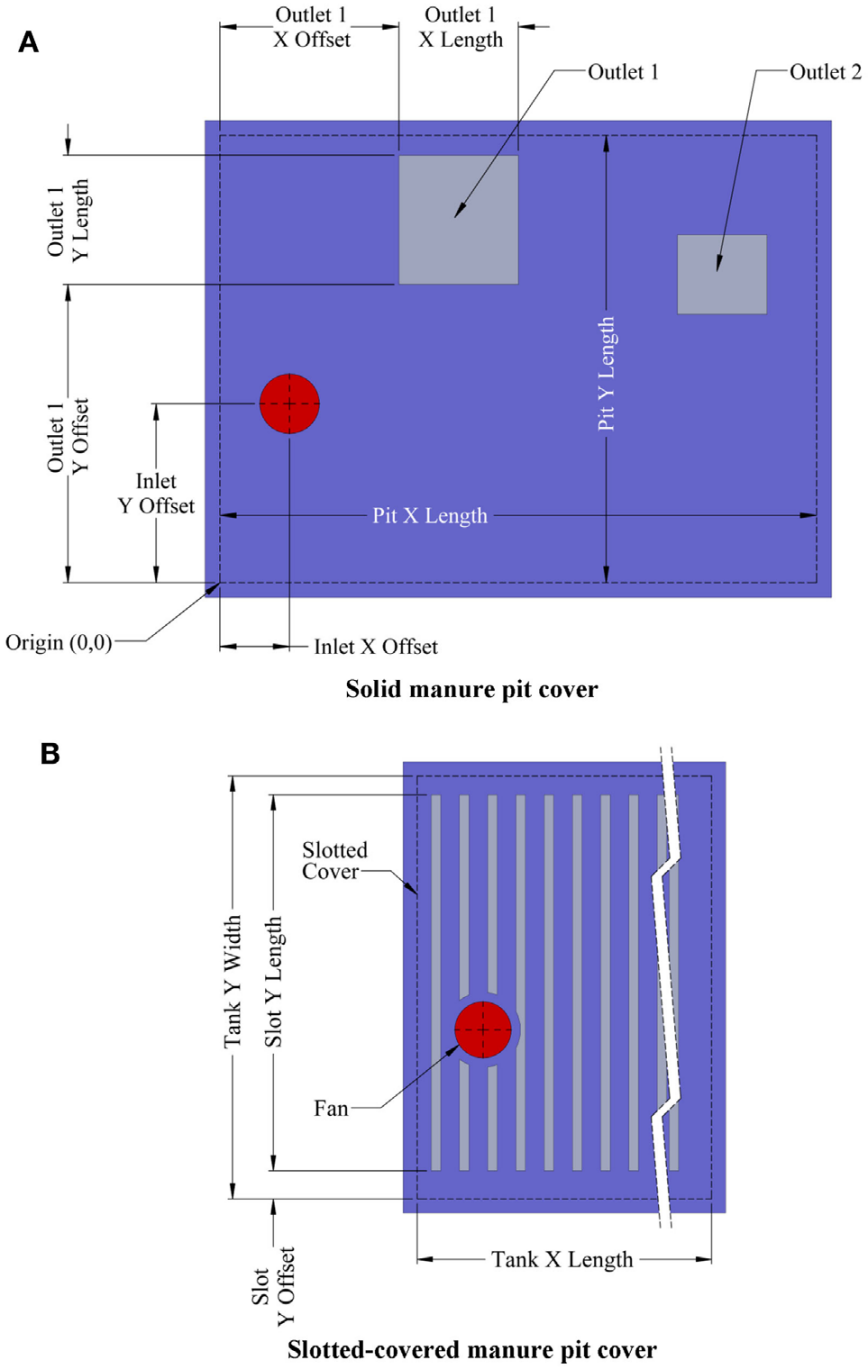
**Definition sketches for inputs for (A) solid manure pit covers and (B) slotted-covered manure pit covers**.

**Figure 6:**
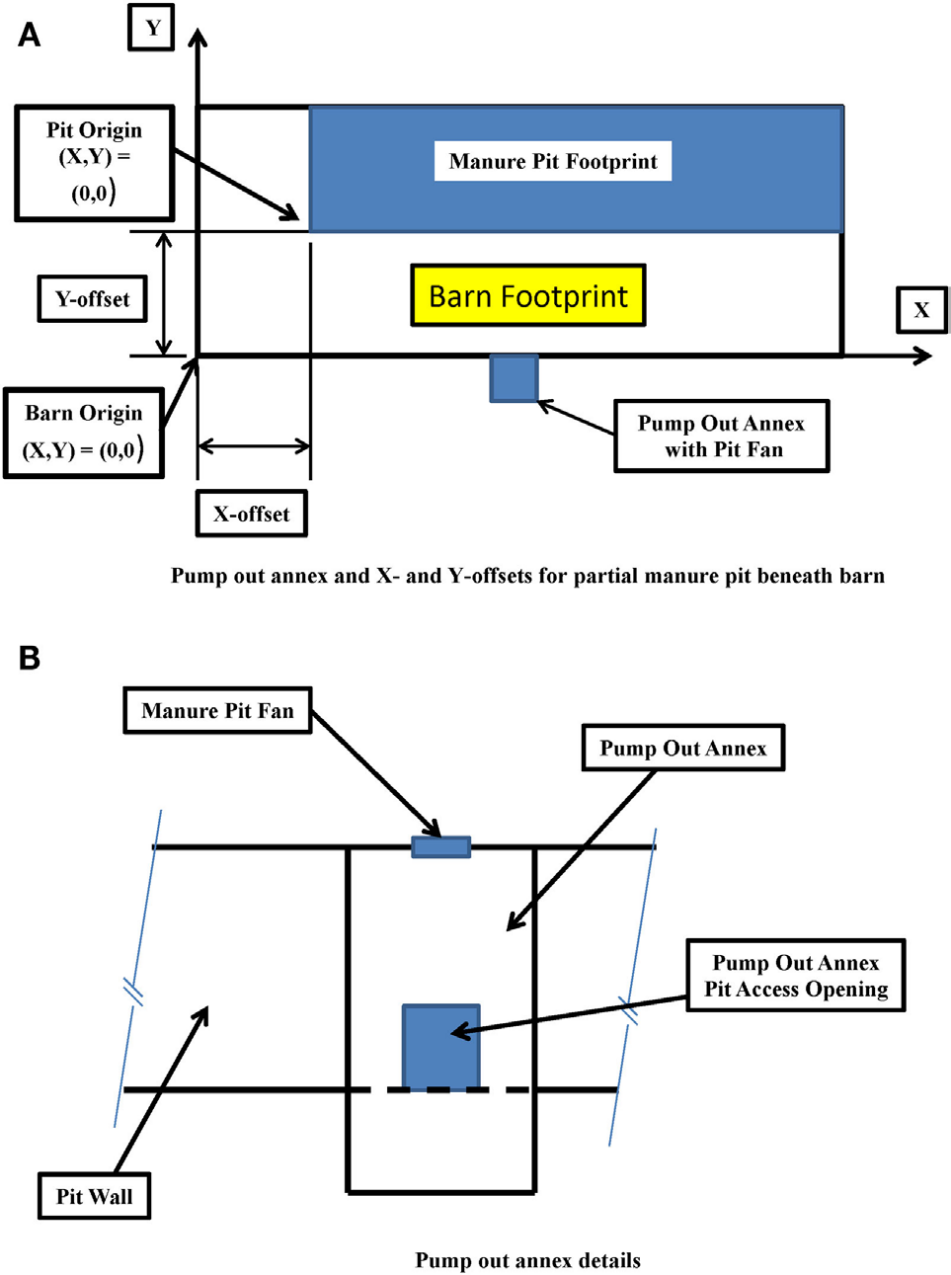
**Definition sketches for (A) typical manure pit pump-out annex and manure pit offsets and (B) pump-out annex details**.

**Figure 7:**
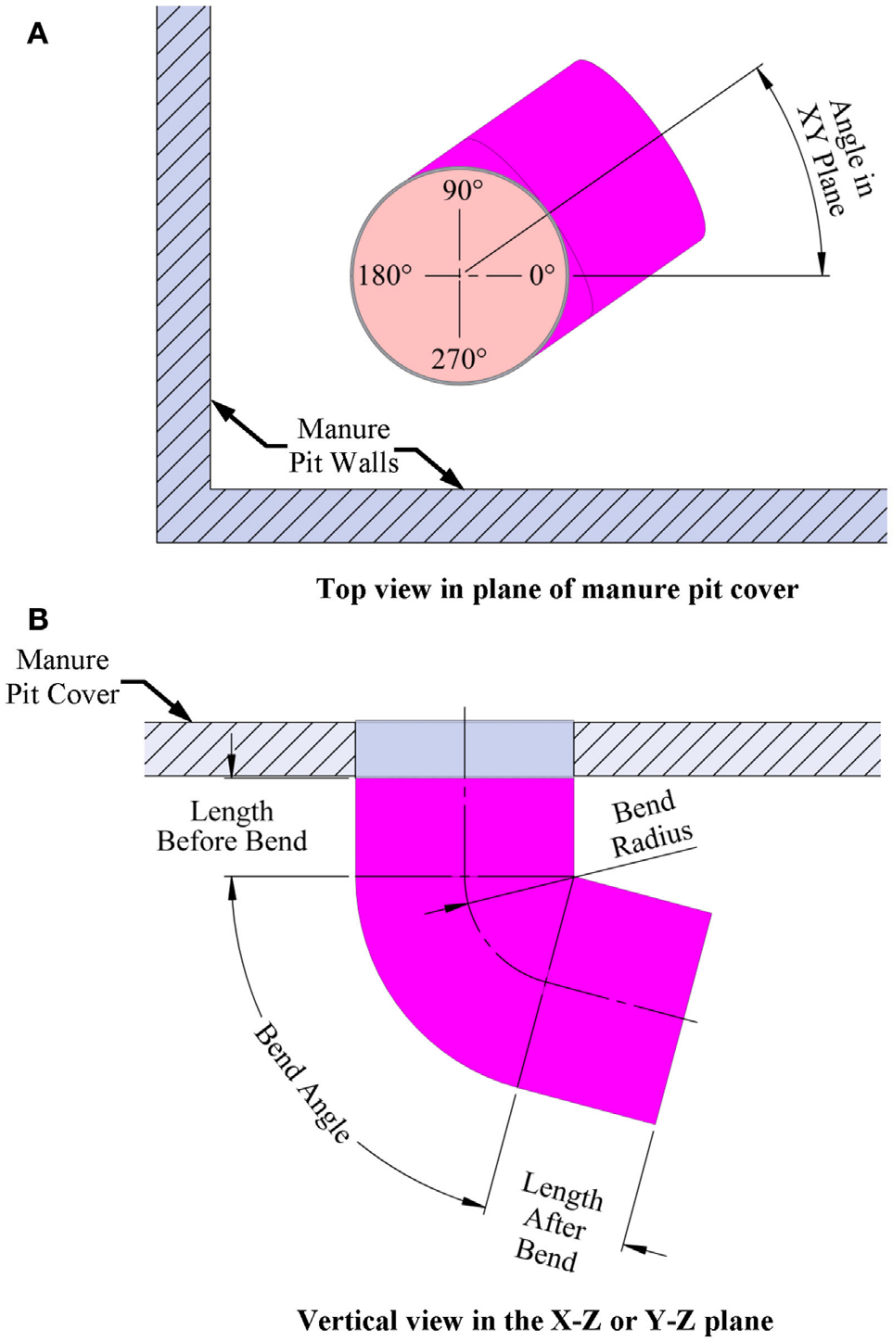
**Definition sketch for pit fan air direction controller: (A) Top view and (B) Side view**.

When in the stand-alone manure pit module, the user is prompted with dialog boxes to enter in turn (Figure 1):

- Pit fan capacity.
- Initial concentration of hydrogen sulfide, carbon dioxide, methane, and ammonia in the manure pit in parts per million (default values of 120 ppm H_2_S, 700 ppm CO_2_, 15,000 ppm CH_4_, and 240 ppm NH_3_ are in the program but can be changed by the user).
- Manure pit cover thickness.
- Manure pit length, width, and height.
- Manure pit ventilation inlet diameter and location defined by *X*- and *Y*-offset distances from the defined Cartesian coordinate system origin (Figure 5A).
- Offset angles for the manure pit ventilation inlet in degrees (if *X*- and *Y*-offsets for the inlet location are positive, both the *X*- and *Y*-offset angle is 90°; if *X*-offset distance is negative, the *X*-offset angle is 270°; and if *Y*-offset is negative, then *Y*-offset angle is 270°).
- Manure pit cover details including whether cover is solid or slotted. A solid cover is the default mode in all modules. For slotted covers, the slotted cover option is selected. Then, the user can define the slotted cover domain (partial or totally slotted) by indicating the *X*- and *Y*-offset distances to the beginning of each slotted cover area, the length and width of each slotted floor domain, the width of slats in each domain, and the width of the slots between slats in each slotted cover domain (Figure 5B).
- Dimensions and *X*- and *Y*-offset distances of up to two manure pit ventilation air outlets for solid covered manure pits. The slots of the covers serve as the manure pit ventilation outlets for slotted-covered manure pits (Figure 5A).
- Location, defined again by *X*- and *Y*-offset distances, and lengths of any longitudinal or transverse divider walls in the manure pit (Figure 1B).
- Location and size (width and height) of up to three perforations in each manure pit divider wall (Figure 1B).
- Locations (*X*- and *Y*-offsets) and dimensions (length, width, and height) of any obstructions to airflow inside the manure pit. A sand mound resulting from the use of sand for bedding is and example of such an obstruction to airflow in the manure pit (Figure 1B).
- Location (*X*- and *Y*-offset distances and *X*- and *Y*-offset angles) and dimensions (length, width, and height) of any manure pit pump-out annexes (Figure 6A). Annex inputs also specify the size and location of the pit wall opening to the annex and the option to place the manure pit fan in a pump-out annex (Figure 6B).
- Source of manure pit ventilation air. The source can be either (1) recirculated air from directly above the manure pit cover or (2) fresh non-contaminated atmospheric air ducted from a location removed from the manure pit cover.
- Direction of manure pit fan airflow into the pit. The air flow can be directed vertically downward into the pit or it can be directed at any vertical angle from horizontal or at any horizontal angle measured from the manure pit longitudinal axis (Figures 7A,B).

#### Checks, Balances, and Job Submission

At any point during the input process, the user can check the input for errors by clicking onto the “Check Constraints” or the “Preview” buttons. The “Check Constraints” button activates a set of equations that checks if geometric entities fall outside of prescribed domains. For example, if a solid-covered pit ventilation outlet falls outside the geometrical domain of the manure pit, the Check Equations identifies this error and sends back an error message. Checking the “Preview” button generates a 3-D rotatable sketch of the manure pit, complete with pit ventilation fan and outlet locations, pump-out annex locations, and other geometric features. This feature helps the user quickly identify geometric and ventilation system input errors. Once the checks have been conducted and any required corrections made, the user clicks the “Save Study” button. This sends the input file to the host server, where it is converted into a format compatible with SWFS^®^ and the simulation is run. The user is informed by email when the simulation is completed and the results are available. At this time, the user again accesses the online tool and follows the prompts to access the simulation results.

#### Inputs for Slotted-Covered Manure Pits beneath Tunnel-Ventilated Barns

After accessing the design aid as explained in Section “Accessing the Design Aid,” the user first selects the tunnel-ventilated option (Figure 2). Three input navigation buttons (Pit Geometry, Barn Geometry, and Pit + Barn) appear at the top of the next input page. The Pit Geometry button directs the user to the data entry for characterizing the pit geometry and pit ventilation details. All data inputs for slotted-covered manure pits beneath naturally ventilated barns are identical to those listed in Section “Inputs for Stand-Alone Manure Pits” for stand-alone manure pits except those for the manure pit ventilation outlets. The slotted floor openings serve as the manure pit ventilation outlets for slotted-covered pits with barns above them.

To enter data for the barn above the manure pit, the user selects the Barn Geometry navigation button. The user is then prompted with dialog boxes to enter the following barn information in turn:

- Barn total fan capacity;
- Barn length (*X*-direction), width (*Y*-direction), and ceiling height (*Z*-direction);
- Barn ventilation air inlet dimensions in the end wall opposite the ventilation fan locations. The barn fan location is always located in the end wall at which *X* = 0.

To establish where the manure pit is located relative to the barn (i.e., define whether the manure pit extends partially or totally under the barn), the user selects the Pit + Barn navigation button. The user then inputs the *X*- and *Y*-offsets for the manure pit relative to the barn (Figure 6A).

The same checks and balances described in Section “Checks, Balances, and Job Submission” are available at any stage of data input for the manure pit beneath tunnel-ventilated barn cases. These checks and balances are available when in any of the three input phases (Pit Geometry, Barn Geometry, or Pit + Barn). Once satisfied with the accuracy of the input data, the user saves the input file and submits it for CFD simulation of the manure pit ventilation and contaminant gas evacuation.

#### Inputs for Slotted-Covered Manure Pits beneath Cross-Ventilated Barns

After accessing the design aid as explained in Section “Accessing the Design Aid,” the user first selects the cross-ventilated option (Figure 3). With the three previously described (see Inputs for Slotted-Covered Manure Pits beneath Tunnel-Ventilated Barns) input navigation buttons (Pit Geometry, Barn Geometry, and Pit + Barn), the user inputs in turn the required manure pit, barn, and barn + pit geometrical and ventilation system data. All data inputs for slotted-covered manure pits beneath cross-ventilated barns are identical to those listed in Section “Inputs for Stand-Alone Manure Pits” for stand-alone manure pits except those for the manure pit ventilation outlets. The slotted floor openings serve as the manure pit ventilation outlets for slotted-covered pits with barns above them.

To enter data for the cross-ventilated barn above the manure pit, the user selects the Barn Geometry navigation button. The user is then prompted with dialog boxes to enter the following barn information in turn:

- Barn length (*X*-direction), width (*Y*-direction), and eave height (*Z*-direction);
- Select the sidewalls or end walls in which barn fans are located;
- Total fan capacity of all fans in each sidewall or end wall;
- Number of fans in each sidewall or end wall;
- Diameter of fans in each sidewall or end wall;
- *X*- or *Y*-offset distance for first fan in each sidewall or end wall (Figure 3A);
- *Z*-offset distance for all fans in each sidewall or end wall;
- Spacing of multiple fans in each sidewall or end wall;
- Length and width of eave ventilation air inlets in each sidewall and end wall;
- Eave inlet blocking locations in each sidewall or end wall;
- Location and dimensions of any barn inlet ducts or drop inlets located between the barn sidewalls (Figure 3B) (these inlets are sometimes used in animal facilities wider than 12–13 m).

The user navigates to the Pit + Barn option to properly locate the manure pit beneath the barn. This input process is identical to that described in Section “Inputs for Slotted-Covered Manure Pits beneath Tunnel-Ventilated Barns.” The user has access to the same checks and balances described in Section “Checks, Balances, and Job Submission” prior to saving and submitting the project for CFD simulation.

#### Inputs for Slotted-Covered Manure Pits beneath Naturally Ventilated Barns

Two additional assumptions are imposed for simulating the ventilation of manure pits beneath naturally ventilated barns (Figure 4). The wind velocity is assumed to be 2.3 m/s. The wind direction is assumed to be plus or minus 45° from the direction perpendicular to the barn sidewalls (Figure 4B). The CFD simulation produces contaminant gas evacuation results for both wind directions.

After accessing the design aid as explained in Section “Accessing the Design Aid,” the user first selects the naturally ventilated option. With the three previously described (see Inputs for Slotted-Covered Manure Pits beneath Tunnel-Ventilated Barns) input navigation buttons (Pit Geometry, Barn Geometry, and Pit + Barn), the user inputs in turn the required manure pit, barn, and barn + pit geometrical and ventilation system data. All data inputs for slotted-covered manure pits beneath naturally ventilated barns are identical to those listed in Section “Inputs for Stand-Alone Manure Pits” for stand-alone manure pits except those for the manure pit ventilation outlets. The slotted floor openings serve as the manure pit ventilation outlets for slotted-covered pits with barns above them.

To enter data for the naturally ventilated barn above the manure pit, the user selects the Barn Geometry navigation button. The user is then prompted with dialog boxes to enter the following barn information in turn:

- Barn roof eave and ridge heights (Figure 4A);
- Barn length (*X*-direction) and width (*Y*-direction);
- Curtain sidewall ventilation opening dimensions (Figure 4A);
- End wall openings: gable sheathing (yes or no); end wall sheathing (yes or no); and dimensions of up to five openings in sheathed end walls (Figure 4B);
- Continuous ridge ventilation air outlet width (Figure 4A);
- Location and size of natural ventilation flow obstructions on each sidewall and end wall (Figure 4B).

The user navigates to the Pit + Barn option to properly locate the manure pit beneath the barn. This input process is identical to that described in Section “Inputs for Slotted-Covered Manure Pits beneath Tunnel-Ventilated Barns.” The user has access to the same checks and balances described in Section “Checks, Balances, and Job Submission” prior to saving and submitting the project for CFD simulation.

## Design Aid Simulation Results

### Results Generated by Design Aid

**Figure 8:**
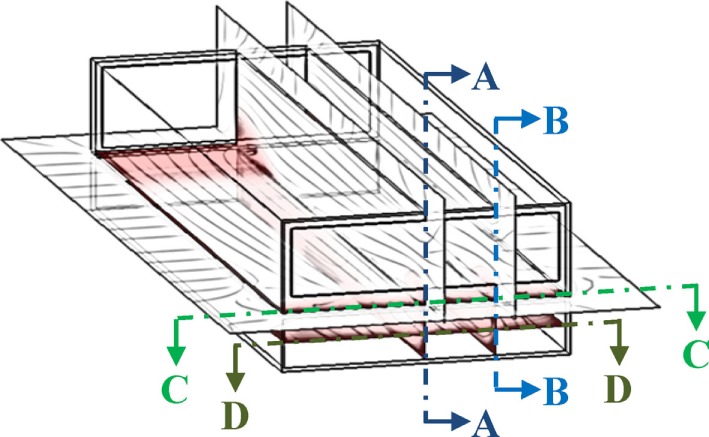
**Definition of animation barn + pit cut-plot locations: vertical longitudinal section through center line (A–A); vertical longitudinal section at quarter point (B–B); horizontal section 152 mm above manure pit cover (C–C); and horizontal section at manure pit mid-height**.

**Figure 9:**
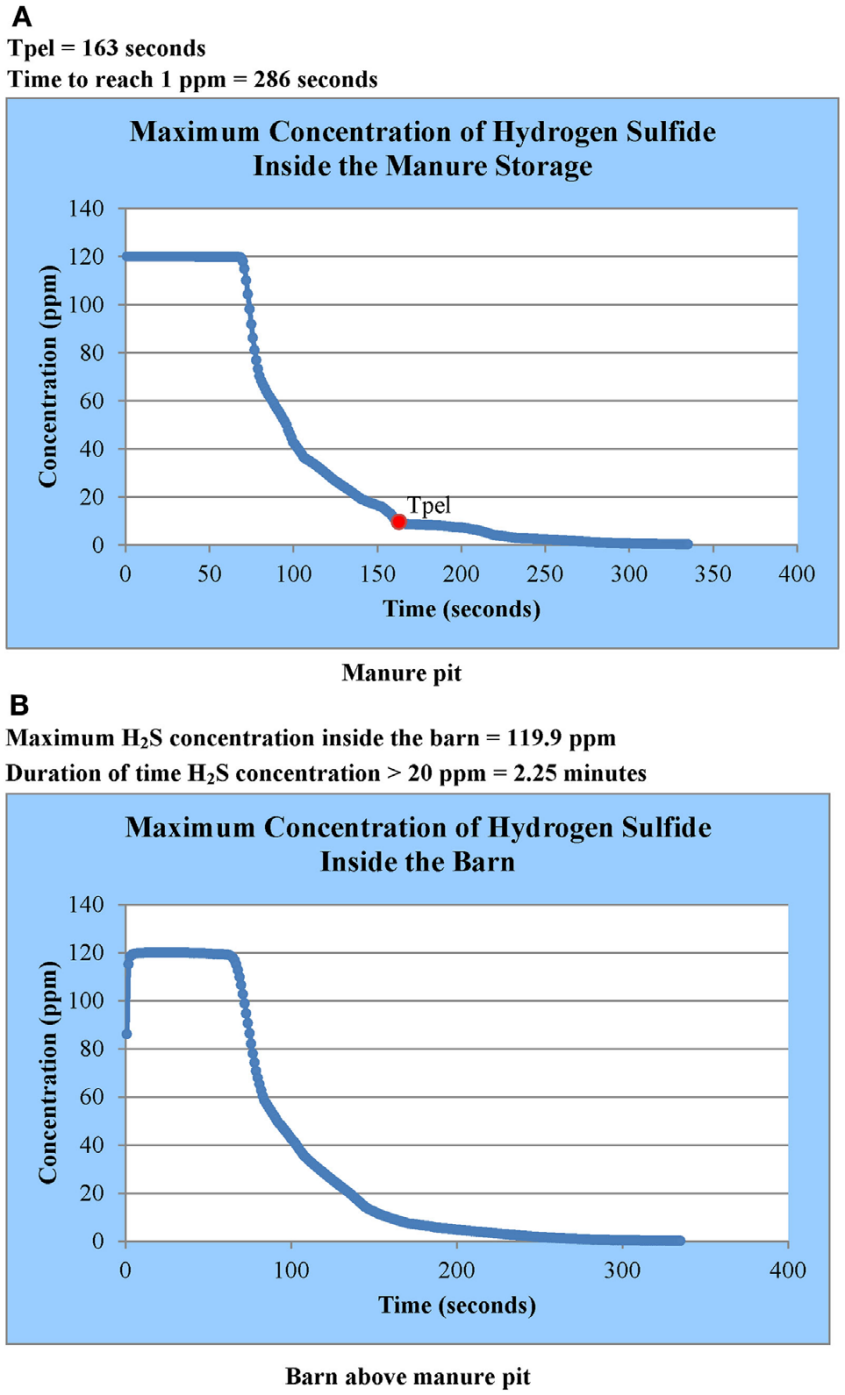
**Hydrogen sulfide decay curves in (A) manure pit and (B) barn above the manure pit for Case Study 1: slotted-covered manure pit beneath a tunnel-ventilated (parallel flow) barn**.

**Figure 10:**
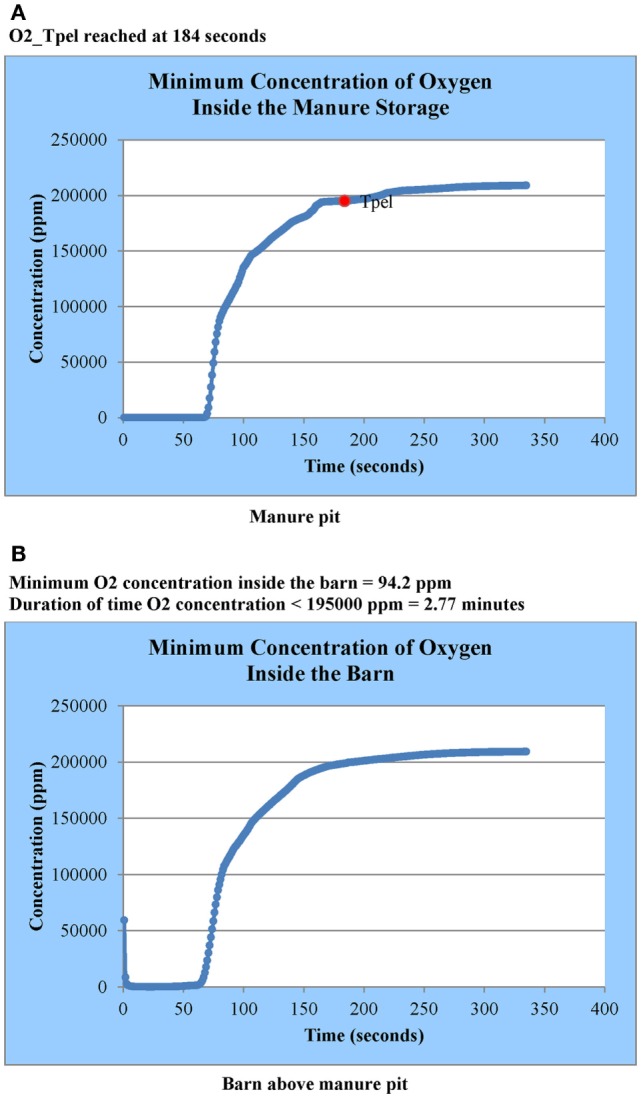
**Oxygen replenishment curves in (A) manure pit and (B) barn above the manure pit for Case Study 1: slotted-covered manure pit beneath a tunnel-ventilated (parallel flow) barn**.

The user is informed by email when SWFS^®^ simulation results are available. This notification may occur within a few hours to a few days depending upon the size project and the number of projects submitted by other users at the same time. Simulation results are accessed by going to the design aid website identified in Section “Accessing the Design Aid” and selecting the project results desired (a user might have several submitted projects at any given time). Project-specific simulation results available to the user are (1) animations of contaminant gas concentrations as a function of manure pit ventilation time for several horizontal and vertical cross-sections (cut plots) in the manure pit and attached barn (Figure 8), (2) two-dimensional plots of maximum contaminant gas concentration inside the manure pit as a function of manure pit ventilation time (Figure 9A), (3) two-dimensional plots of maximum contaminant gas concentration inside attached barns as a function of manure pit ventilation time (Figure 9B), (4) two-dimensional plots of minimum oxygen concentration inside the manure pit as a function of manure pit ventilation time (Figure 10A), and (5) two-dimensional plots of minimum oxygen concentration inside attached barns as a function of manure pit ventilation time (Figure 10B). Two sets of animations and gas concentration decay or oxygen replenishment plots are provided for cases with naturally ventilated barns above slotted-covered manure pits: one for wind direction oriented 45° clockwise from the transverse building axis and the other for wind direction oriented 45° counterclockwise from the transverse building axis (Figure 4B).

**Figure 11:**
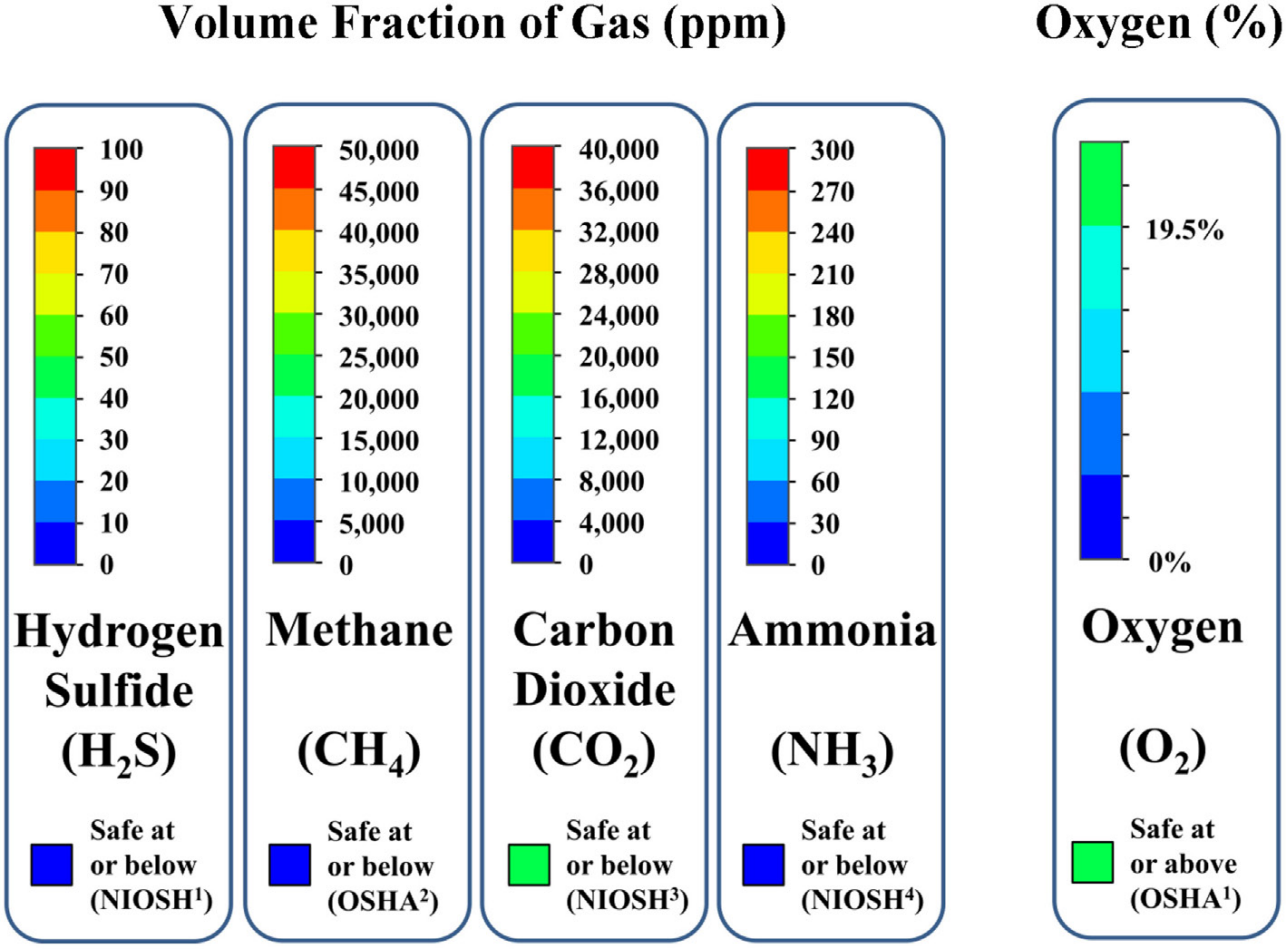
**Color code legends for interpreting animations of gas concentrations in the manure pit or barn as function of time from commencement of pit ventilation**. Red indicates that contaminant gas concentration above potentially lethal limit; blue indicates that contaminant gas concentration is below its TLV or PEL. Green indicates that oxygen levels are 19.5% or higher; blue indicates that oxygen level of 0% by volume.

The contaminant gas and oxygen concentration animations are available for the horizontal and vertical cross-sections that are identified in Figure 8. Animations of gas concentration decay for several transverse vertical cross-sections are provided for cross-ventilated barns above slotted-covered manure pits. All animations are color coded per gas concentration level and uniquely so for each gas simulated (i.e., the color code for 1,000-ppm concentration is different for each gas). Figure 11 presents the color code legend for each gas simulated. This gas-specific color coding was selected because of the large differences in typical concentrations of each gas in manure pits.

The plots of manure pit maximum gas concentration vs. ventilation time identify the pit ventilation time required to evacuate a contaminant gas concentration anywhere in the pit to below OSHA-defined PELs or ACGIH-defined TLVs, for example, the pit ventilation time required to reduce hydrogen sulfide concentration in the manure pit from the initial pit concentration to either OSHA-defined (2) PEL concentration of 10 ppm or ACGIH-defined (3) concentration of 1 ppm (Figure 9A). The PELs or TLVs for determining required pit ventilation times for long-term human occupancy are 1,000, 5,000, and 25 ppm, respectively, for methane, carbon dioxide, and ammonia (2, 3).

The plots of attached barn maximum gas concentration vs. ventilation time identify the maximum concentration of contaminant gases in the attached barn and the length of time the maximum contaminant gas concentration anywhere in the barn exceeds the gas-specific, short-term exposure limit (STEL) or short-time exposure ceiling limit. For example, the length of time the maximum hydrogen sulfide concentration exceeds either the OSHA-defined (2) short-time exposure ceiling limits of 20 ppm if there has been some prior hydrogen sulfide exposure or 50 ppm if there has been no prior hydrogen sulfide exposure (Figure 9B). The STELs selected in the design aid for evaluating the need to evacuate parts, or all, of the barn are, respectively, 25,000, 20,000, and 35 ppm for methane, carbon dioxide, and ammonia. The selected STEL for methane is 50% of the lower explosive limit (LEL). The selected STEL for carbon dioxide (20,000 ppm) is more conservative than the time-weighted average value (30,000 ppm). The selected STEL for ammonia (35 ppm) is more conservative than the OSHA-defined (2) TWA of 50 ppm. The user can adopt more or less conservative short-term exposure criteria for evacuation of all or portions of the barn by using the respective contaminant gas concentration decay curves and barn cut-plot animations.

The plots of oxygen replenishment during ventilation similarly identify the pit ventilation time required to increase oxygen concentration anywhere in the pit to the ACGIH-defined TLV of 19.5% by volume. The duration of time that oxygen levels anywhere in the barn are less than 19.5% by volume is specifically identified and highlighted (Figures 10A,B).

### Applying Design Aid Results

For both stand-alone and slotted-covered manure pits beneath barns, the design aid results identify the required pit ventilation time to evacuate contaminant gases anywhere in the pit to below regulatory agency-defined levels for short-term and long-term human occupancy. It also defines the required pit ventilation time to replenish oxygen levels from 0 ppm to regulatory agency-defined levels for short-and long-term human occupancy. Agricultural building and manure pit designers and planners, as well as agricultural safety specialists and industrial hygienists, can use these design aid results to recommend minimum pit ventilation times and pit fan capacities to apply before human entry into the manure pit. The design aid can also be used to explore alternative pit ventilation design details – such as fan capacity, fan location, pit ventilation air outlet location, and the source of pit ventilation air (above pit or from a contaminant free area) for both temporary portable or permanent pit ventilation system applications.

For slotted-covered manure pits beneath any type of ventilated barn, the design aid results can be used to determine if any portion, or all, of the barn needs to be evacuated during a manure pit ventilation event. In addition, the design aid can be useful for evaluating alternative manure pit ventilation system configurations that minimize the degree of manure gas contamination in the barn during manure pit ventilation. For example, the designer might examine the effect of decreasing the manure pit ventilation rate, thereby increasing the time required to ventilate the pit, but reducing the proportion of the barn from which animals and personnel need to be evacuated. Or, the designer might alter the location of the manure pit ventilation fan to determine the best location for reduction of the contaminated zone in the barn.

**Figure 12:**
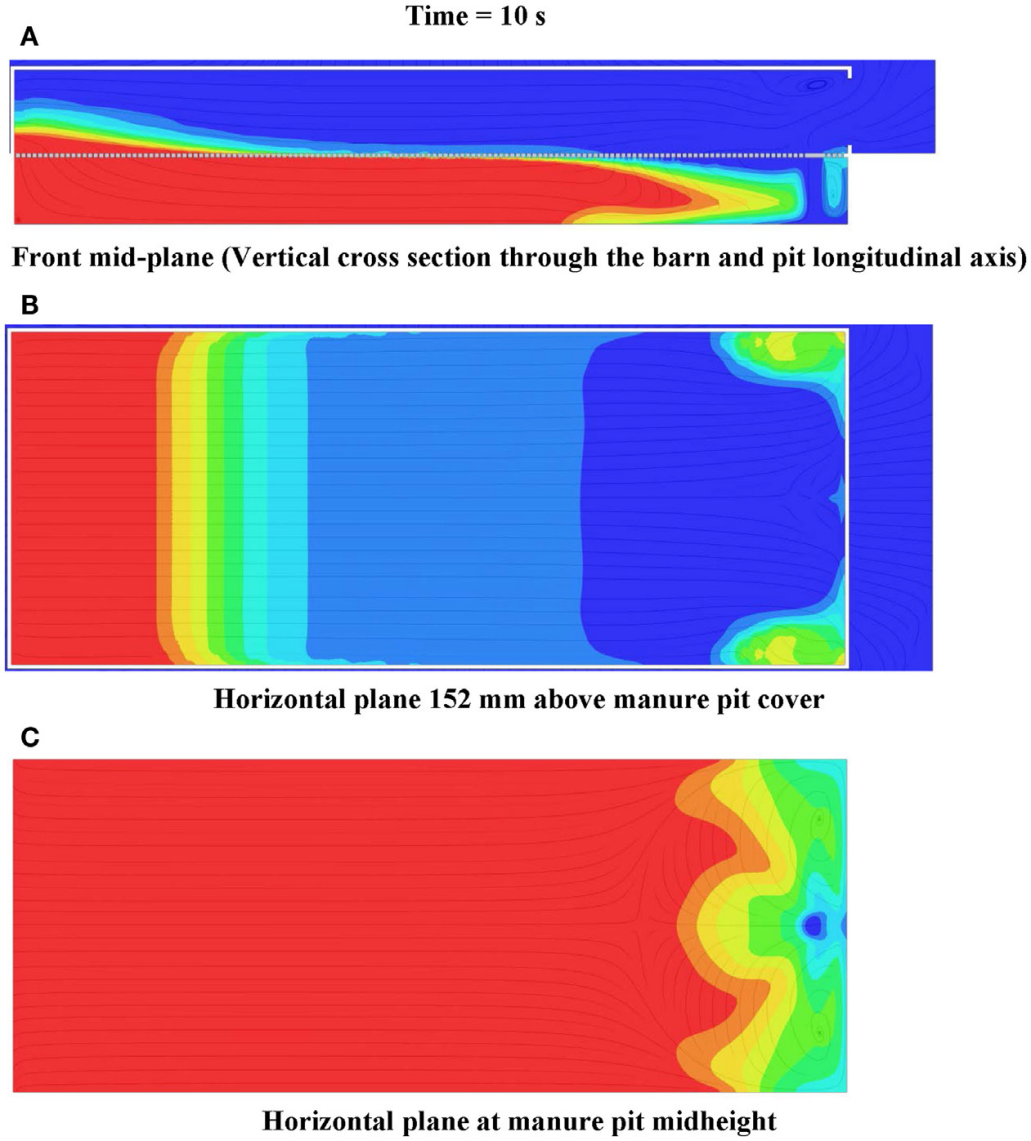
**Frames of (A) front mid-plane, (B) horizontal plane 152 mm above manure pit cover, and (C) horizontal plane at manure pit mid-height cut plot animations of hydrogen sulfide concentration 10 s after commencement of pit ventilation for Case Study 1: tunnel ventilated (parallel flow) barn above slotted-covered manure pit (see Figure 11 for color legend for hydrogen sulfide)**.

A multiple-step evaluation of the simulation results is necessary to determine which portions, if any, of the barn needs to be evacuated prior to a manure pit ventilation event: (1) use the barn contaminant gas decay curves (Figure 9B) to determine if the maximum contaminant gas concentration exceeds the STEL, short-term ceiling limit, or other defined limiting concentration level, anywhere in the barn; (2) use the horizontal cross-section 150 mm above the slotted cover animations to determine which portions of the barn reach STEL, or other defined limiting levels, during the pit ventilation event (e.g., Figure 12B); and (3) use the vertical cross-section animations to determine the height of the zone, in which limiting gas concentrations are exceeded during the pit ventilation event (e.g., Figure 12A). Using this stepwise examination of the design aid results, the designer or safety specialist is able to make an informed decision about the degree of personnel and animal evacuations required prior to a manure pit ventilating event.

## Case Studies

Two case studies are presented. The first is for a slotted-covered manure pit beneath a tunnel-ventilated barn with two alternative manure pit ventilation configurations. The second is for a slotted floor covered manure pit beneath a mechanically cross-ventilated barn. Both case studies represent a typical manure pit beneath a fully stocked swine finishing barn.

### Case Study 1: Slotted-Covered Manure Pit beneath a Tunnel-Ventilated Barn

The manure pit ventilation and gas evacuation simulation results for a slotted-covered manure pit beneath a tunnel-ventilated barn are now presented for two manure pit ventilation configurations (Figure 2). These results first illustrate simulation results for the two ventilation system. Then, the results are used to compare the performance of two alternative manure pit strategies to decide which one is the better option.

#### Case 1 Description – Parallel and Counter Flow

This case study is for a 12.2-m wide by 30.5-m long by 3.05-m ceiling height swine barn for 450 finishing pigs above a totally slotted-covered 12.2-m wide by 30.5-m long by 2.44-m deep manure pit. The barn negative pressure ventilation capacity, 226.0 m^3^/s, is the design hot weather ventilation rate for 450 finishing pigs (9, 15). Initial manure pit gas concentrations are 120, 85,000, 70,000, 300, and 0 ppm, respectively, for hydrogen sulfide, methane, carbon dioxide, ammonia, and oxygen.

The manure pit is ventilated with a 610-mm diameter, 4.5 m^3^/s pit fan located 6.10 m from the sidewalls and 610 mm from either the end wall containing the barn ventilation exhaust fans or the end wall containing the barn ventilation air inlets. The first pit fan location is characterized as parallel flow (Figure 2B), and the latter as counter flow (Figure 2C). In both air flow cases, the pit fan directs recirculated barn air from directly above the slotted-cover into the pit perpendicular to the slotted-cover plane.

#### Hydrogen Sulfide Decay Concentration Curves (Pit and Barn) – Parallel Flow

Figure 9A is the simulated hydrogen sulfide decay curve for the manure pit for the parallel flow case. The decay curve is not for a particular location in the pit. Instead, it is a plot of the maximum concentration anywhere in the manure pit domain as a function of manure pit ventilation time. The legend in Figure 9A identifies and Table 1 lists the ventilation time required to reduce the maximum pit hydrogen sulfide concentration to either the OSHA-defined level of 10 ppm (163 s) or the ACGIH-defined level of 1 ppm (286 s).

**Table 1:** **Case 1 simulation results for hydrogen sulfide, methane, carbon dioxide, and ammonia: slotted-covered manure pit beneath tunnel-ventilated barn – parallel flow**.

Gas	Initial pit gas concentration (ppm)	Pit ventilation time to reach TLV or PEL^a^ (s)	Maximum gas concentration in barn (ppm)	Pit ventilation time to reach limiting gas concentration in barn^b^ (s)
Hydrogen sulfide	120	163 (10 ppm)	119	135
		286 (1 ppm)		
Methane	85,000	275	84,962	109
Carbon dioxide	70,000	174	69,969	109
Ammonia	300	163	300	145

*^a^PEL for hydrogen sulfide is either 1 or 10 ppm; TLV for methane is 1,000 ppm; PEL for carbon dioxide is 5,000 ppm; and TLV for ammonia is 25 ppm (2, 3)*.

*^b^Short-term ceiling limit for hydrogen sulfide is 20 ppm (2); investigator-defined limiting concentration is 25,000 ppm for methane, 20,000 ppm for carbon dioxide, and 25 ppm for ammonia (see Results Generated by Design Aid)*.

Figure 9B is the simulated hydrogen sulfide decay curve for the barn for the parallel flow case. Again, the decay curve shows the maximum hydrogen sulfide concentration in the barn domain as a function of manure pit ventilation time. The figure legend identifies and Table 1 lists the maximum hydrogen sulfide level anywhere in the barn domain and the time period that the maximum hydrogen sulfide is above the OSHA-defined ceiling limit of 20 ppm (135 s) (2) The decay curves do not identify the zones of the barn for which hydrogen sulfide concentrations exceed the ceiling limit. The barn contamination zones are identified by examination of the cut-plot animation of hydrogen sulfide concentrations through the horizontal plane 152 mm above the slotted cover.

#### Hydrogen Sulfide Cut Plots – Parallel Flow

Figure 12 shows the frames of three hydrogen sulfide concentration animations 10 s after the commencement of pit ventilation. The animations are for, respectively, the vertical plane through the barn and pit longitudinal axis (Figure 12A), the horizontal plane 150 mm above the slotted cover (Figure 12B), and the horizontal plane located at the pit mid-height (Figure 12C). These frames clearly identify the zones (those colored zones ranging from light blue to red), in which hydrogen sulfide concentrations exceed the 20-ppm ceiling exposure limit during pit ventilation. For the case study barn and parallel flow ventilation configuration, the zone of barn contamination exceeding 20 ppm is confined to one located within 10.8 m of the end wall containing the barn exhaust ventilation fans. If the less conservative short-term ceiling exposure limit of 50 ppm is selected, only those barn zones within 6.1 m (those colored zones ranging from green to red) of the end wall containing the barn fans need to be evacuated (Figure 12B).

#### Oxygen Replenishment Curves in (Pit and Barn) – Parallel Flow

Figure 10A is the simulated oxygen replenishment curve for the manure pit for the parallel flow case. The replenishment curve is not for a particular location in the pit. Instead, it represents the minimum concentration anywhere in the manure pit domain as a function of manure pit ventilation time. The legend identifies the ventilation time required to replenish pit oxygen concentration in the entire pit to the ACGIH-defined TLV of 19.5% (184 s).

Figure 10B is the simulated oxygen replenishment curve for the barn for the parallel flow case. Again, the plot shows the minimum oxygen concentration in the barn domain as a function of manure pit ventilation time. The figure legend identifies the minimum oxygen concentration level anywhere in the barn domain and the time period that the minimum oxygen concentration is less than 19.5% by volume (166 s). The replenishment curves do not identify the zones of the barn for which oxygen concentrations are less than 19.5%. These zones are identified by examination of the horizontal barn cut-plot animations of oxygen concentration as described in Section “Hydrogen Sulfide Cut Plots – Parallel Flow” for hydrogen sulfide.

#### Simulation Results for Other Contaminant Gases (Pit and Barn) – Parallel Flow

Table 1 reports the simulation results for all contaminant gases for the parallel flow case study. Simulation results for methane, carbon dioxide, and ammonia were obtained from gas decay curves similar to those presented in Section “Hydrogen Sulfide Decay Concentration Curves (Pit and Barn) – Parallel Flow” for hydrogen sulfide.

Simulated ventilation times to evacuate contaminant gases in the manure pit to below PELs or TLVs for the case study geometry, ventilation configuration, and ventilation rates are 275, 174, and 163 s, respectively, for methane, carbon dioxide, and ammonia. Maximum barn contaminant gas concentrations approach the initial concentrations somewhere in the barn during the first few seconds after commencement of pit ventilation. Methane, carbon dioxide, and ammonia concentrations in the barn exceed the previously defined short-term limiting concentrations for 109, 109, and 145 s, respectively. The horizontal and vertical cut-plot animations of contaminant gas concentrations in the barn show a pattern almost identical to that shown earlier for hydrogen sulfide. The barn zone within approximately 10.8 m of the end wall containing the barn exhaust fans is the only one with contaminant gas concentrations exceeding the short-term limits.

#### Selected Simulation Results for Tunnel-Ventilated Case Study with Counter Flow Ventilation

**Figure 13:**
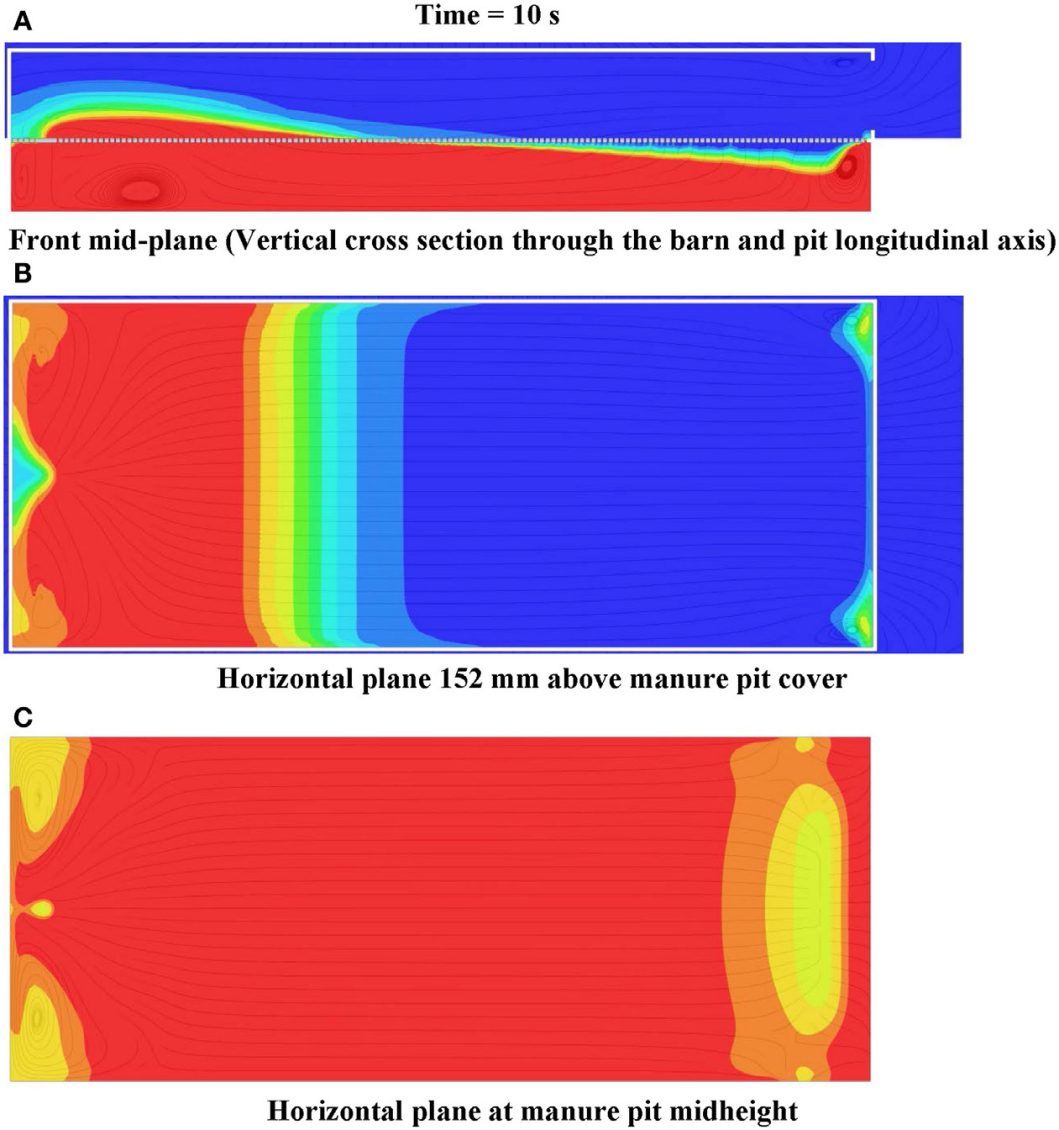
**Frames of (A) front mid-plane, (B) horizontal plane 152 mm above manure pit cover, and (C) horizontal plane at manure pit mid-height cut plot animations of hydrogen sulfide concentration 10 s after commencement of pit ventilation for Case Study 1: tunnel ventilated (counter flow) barn above slotted-covered manure pit (see Figure 11 for color legend for hydrogen sulfide)**.

Only the results for hydrogen sulfide are presented for the slotted-covered manure pit beneath a counter flow ventilated (Figure 2C) barn. The required pit ventilation times to evacuate hydrogen sulfide to 10 and 1 ppm, taken from the simulated manure pit gas decay curve, are 510 and >737 s, respectively. The design aid sometimes terminates the simulation when, as in this case, the hydrogen sulfide manure pit concentration approaches 1 ppm nearly asymptotically. This avoids excessive computer run time. Examination of the hydrogen sulfide decay curve for this case study example clearly showed that a pit ventilation time of approximately 750 s is a satisfactory ventilation time for evacuation of hydrogen sulfide in the pit to approximately 1 ppm. From the simulated barn gas decay curve, hydrogen sulfide concentrations in the barn exceed the OSHA-defined (2006) ceiling limit of 20 ppm for 315 s. Figures 13A-C are frames from three animations of hydrogen sulfide concentrations in the pit and barn 10 s after commencement of pit ventilation. Figure 13A is for a vertical cross-section through the barn and pit longitudinal centerline; Figure 13B is for the horizontal plane 150 mm above the slotted cover; Figure 13C is for the horizontal plane at the pit mid-height. The frames clearly show (the colored zone ranging from light blue to red) that the barn zones in which hydrogen sulfide concentration is greater than 20 ppm extend 12.2 m from the end wall containing the barn ventilation fans. If the less conservative ceiling limit of 50 ppm is selected, only those barn zones within 10.5 m (those colored zones ranging from green to red) of the end wall containing the barn fans need to be evacuated (Figure 13B).

The ventilation times to evacuate hydrogen sulfide to 10 and 1 ppm in the manure pit are 312% (510 vs. 163 s) and more than 257% (>737 vs. 286 s) greater, respectively. The time period during which hydrogen sulfide concentrations in the barn exceed 20 ppm are 233% (315 vs. 135 s) greater for counter flow ventilation compared to the parallel flow manure pit ventilation configuration. The zone in which barn contamination exceeds the OSHA-defined short-term ceiling limit of 20 ppm is also 12% larger (12.1 vs. 10.8 m from the end wall containing the barn fans) for the counter flow configuration compared to the parallel flow configuration. The design aid shows that the parallel flow configuration is clearly the better one for ventilating this manure pit and barn.

### Case 2: Slotted-Covered Manure Pit beneath a Cross-Ventilated Barn

Case Study 2 is a simulation of the same 12.2-m wide by 30.5-m long slotted-covered manure pit beneath a barn of the same size used in Case 1 (see Case 1 Description – Parallel and Counter Flow) (Figure 3). The initial manure pit gas concentrations are the same except initial methane and carbon dioxide concentrations are 70,000 and 85,000 ppm, respectively. The pit fan size and capacity, and the source and direction of manure pit ventilation air are identical to Case Study 1. The only differences between Case Studies 1 and 2 are the location of the pit fan and the configuration of the barn ventilation system.

The 610-mm (4.5 m^3^/s) diameter pit fan is located in the slotted floor at a location along the transverse centerline of the barn and offset 1.22 m from the sidewall opposite the barn fans (Figure 3A). The pit fan air supply is taken from directly above the slotted cover; the pit fan directs air downward at an angle 90° from the manure pit cover.

The ventilation system for the cross-vented barn consists of three identical and uniformly spaced 1.32-m diameter negative pressure fans located on one sidewall and 203-mm wide slotted air inlets located along the entire eave length of both sidewalls. The total AC of the three barn ventilation fans is 26.0 m^3^/s. The first fan is offset 6.10 m from one end wall; the last sidewall fan is offset 6.1 m from the other end wall.

#### Contaminant Gas Concentration Decay and Oxygen Replenishment Curve Results: Cross-Ventilated Barn

Table 2 reports the simulation results for all contaminant gases for the cross-ventilated case study. Simulation results for hydrogen sulfide, methane, carbon dioxide, and ammonia were obtained from gas decay curves similar to those presented in Section “Contaminant Gas Concentration Decay and Oxygen Replenishment Curve Results: Cross-Ventilated Barn” for hydrogen sulfide decay in tunnel-ventilated barns (Figures 9A,B). The cross-ventilated decay curves are not shown for brevity.

**Table 2:** **Case 2 simulation results for hydrogen sulfide, methane, carbon dioxide, and ammonia: slotted-covered manure pit beneath cross-ventilated barn**.

Gas	Initial pit gas concentration (ppm)	Pit ventilation time to reach TLV or PEL^a^ (s)	Maximum gas concentration in barn (ppm)	Pit ventilation time to reach limiting gas concentration in barn^b^ (s)
Hydrogen sulfide	120	303 (10 ppm)	119	182
		>549 (1 ppm)		
Methane	70,000	523	69,612	119
Carbon dioxide	85,000	351	84,528	156
Ammonia	300	303	298	223

*^a^PEL for hydrogen sulfide is either 1 or 10 ppm; PEL for methane is 2,500 ppm; PEL for carbon dioxide is 5,000 ppm; and PEL for ammonia is 25 ppm (2, 3)*.

*^b^Short-term ceiling limit for hydrogen sulfide is 20 ppm (2); investigator-defined limiting concentration is 25,000 ppm for methane, 20,000 ppm for carbon dioxide, and 25 ppm for ammonia (see Results Generated by Design Aid)*.

The ventilation time required to reduce the maximum manure pit hydrogen sulfide concentration from 120 ppm to the OSHA-defined level of 10 ppm is 303 s; and the corresponding ventilation time to evacuate hydrogen sulfide to the ACGIH-defined level of 1 ppm is >549 s. The design aid sometimes terminates the simulation when, as in this case, the hydrogen sulfide manure pit concentration approaches 1 ppm nearly asymptotically. This avoids excessive computer run time. Examination of the hydrogen sulfide decay curve for this case study example clearly showed that a pit ventilation time of 540 s is a satisfactory ventilation time for evacuation of hydrogen sulfide in the pit to approximately 1 ppm. Simulated ventilation times to evacuate the remaining manure pit contaminant gas concentrations to below PELs or TLVs for the case study geometry, ventilation configuration, and ventilation rates are 523, 351, and 303 s, respectively, for methane, carbon dioxide, and ammonia.

The maximum contaminant gas concentration in the barn domain as a function of manure pit ventilation time was obtained from contaminant gas decay curves similar to Figure 11B. Table 2 lists the maximum hydrogen sulfide level anywhere in the barn domain and the time period that the maximum hydrogen sulfide is above the OSHA-defined ceiling limit of 20 ppm (182 s). Methane, carbon dioxide, and ammonia concentrations in the barn exceed the previously defined short-term limiting concentrations for 119, 156, and 223 s, respectively. Table 2 also lists the maximum contaminant gas concentrations in the barn during manure pit ventilation. Maximum barn contaminant gas concentrations approach the initial manure pit concentrations somewhere in the barn during the first few seconds after commencement of pit ventilation.

From oxygen replenishment curves similar to those shown in Figure 10A for the Case 1 slotted-covered manure pit beneath a tunnel-ventilated barn, the ventilation time required to replenish oxygen concentration in the entire pit to 19.5% by volume is 327 s. From oxygen replenishment curves similar to those shown in Figure 10B, the time period that the minimum oxygen concentration in the barn is below the ACGIH-defined TLV of 19.5% by volume is 295 s.

The barn contaminant gas decay and oxygen replenishment results alert the user that animals and personnel need to be evacuated from portions of the barn before pit ventilation commences. The cut-plot animations of gas concentration need to be inspected to determine which zones require evacuation.

#### Contaminant Gas Cut-Plot Results: Cross-Ventilated Barn

**Figure 14:**
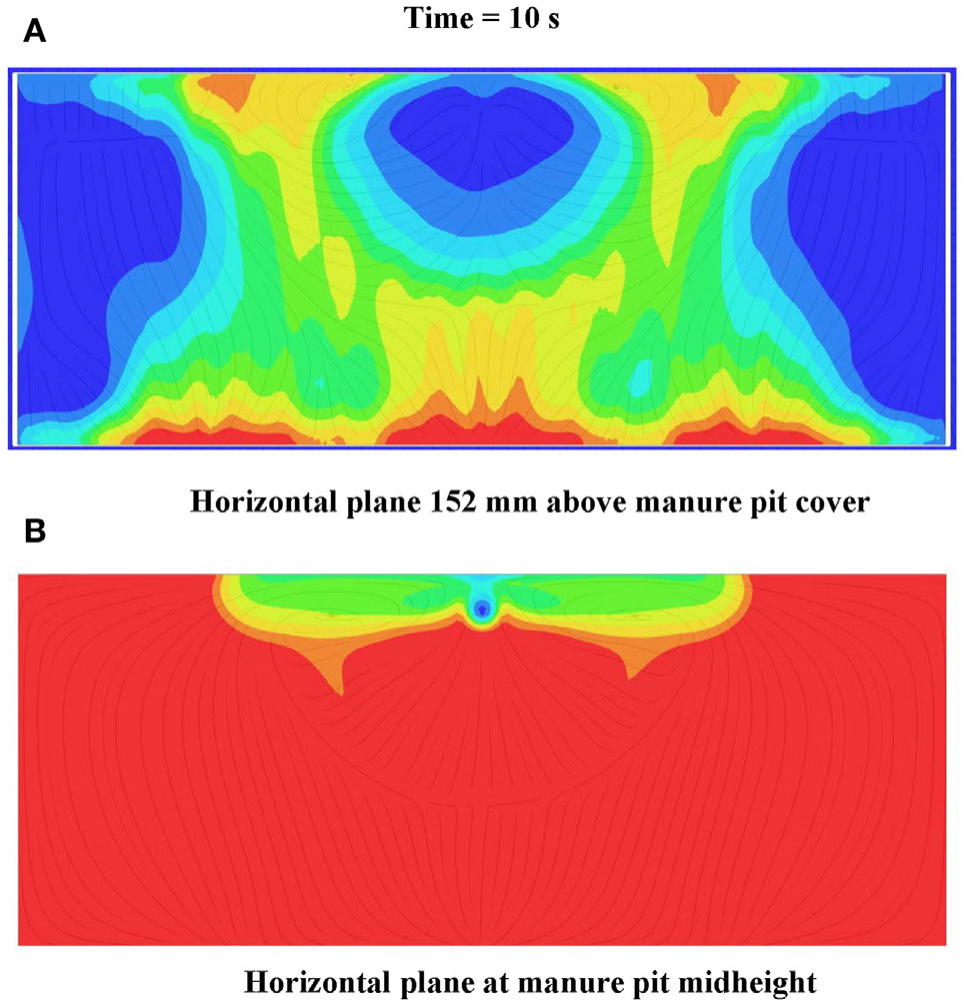
**Frames of (A) horizontal plane 152 mm above manure pit cover, and (B) horizontal plane at manure pit mid-height cut plot animations of hydrogen sulfide concentration 10 s after commencement of pit ventilation for Case Study 2: cross-ventilated barn above slotted-covered manure pit (see Figure 11 for color legend for hydrogen sulfide)**.

**Figure 15:**
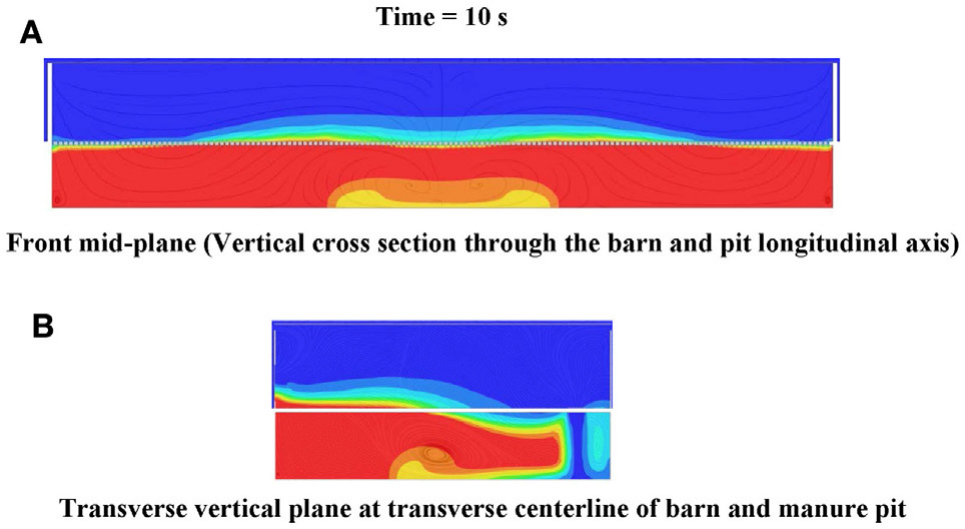
**Frames of (A) front mid-plane and (B) transverse vertical plane at centerline of barn and pit cut plot animations of hydrogen sulfide concentration 10 s after commencement of pit ventilation for Case Study 2: cross-ventilated barn above slotted-covered manure pit (see Figure 11 for color legend for hydrogen sulfide)**.

Figures 14A,B are frames from the simulated hydrogen sulfide animations 10 s after commencement of manure pit ventilation for, respectively, the horizontal plane 150 mm above the slotted cover and the horizontal plan at the manure pit mid-height. Figures 15A,B are similar 10-s animation frames for the vertical plane through the longitudinal centerline of the barn and pit, and the transverse vertical plane at the transverse centerline of the barn and pit. These frames clearly identify the zones (those in light blue to red), in which hydrogen sulfide concentrations exceed the 20-ppm ceiling limit during pit ventilation. For the barn and pit ventilation configuration and initial conditions of the cross-ventilated case study barn and barn cross-ventilated configuration, the zone of barn contamination exceeding the ceiling limit is extensive and scattered throughout the barn footprint and throughout large portions of the vertical barn profile. The horizontal and vertical cut-plot animations of methane, carbon dioxide, and ammonia concentrations in the barn show a pattern almost identical to that shown for hydrogen sulfide. That is, the zone of barn contamination exceeding the previously defined short-term limits is extensive (more than 80% of the total footprint area) and scattered throughout the barn footprint.

#### Case Study 2 Closure

For the case study barn and pit ventilation configuration and initial conditions, animals and personnel need to be evacuated from the entire barn prior to manure pit ventilation. Alternatively, the design professional or regulatory personnel could use the design aid to simulate alternative manure pit and/or barn ventilation strategies. Potential alternative strategies include pit fan location, pit fan air flow direction, pit fan air supply ducted from non-contaminated fresh air source, and strategic blocking of barn ventilation air inlets. If no combination of alternative pit and/or ventilation strategies satisfactorily limits the barn contamination, then the only alternative is to evacuate animals and personnel from the barn before ventilating the manure pit.

## Discussion

The described design aid simulation protocols and results are useful for determining when contaminant gas concentrations have been evacuated from the entire manure pit, or portions thereof, to levels suitable for human entry. The results therefore are useful for defining the portions of the manure pit that can be entered for planned repair and maintenance or for emergency situations even when self-contained breathing equipment is not available to personnel. This is important because few farms have such equipment (16). However, many do have access to fans and blowers for pit ventilation prior to an entry event.

The online tool is a pre-and post-processing software that interfaces typical manure pit and barn configurations and ventilation configurations with modern CFD and CAD software. The tool is particularly useful to users who use CFD software infrequently and cannot justify purchasing software licenses for only a few projects annually. The developed online tool offers a user-friendly, cost-effective alternative for these users.

The online design aid results are not intended to replace the need to continuously monitor confined-space manure pits for contaminant gases and oxygen content prior to and during an entry event. It is recommended that all entry events be conducted by (1) monitoring contaminant manure pit gas and oxygen levels prior to pit ventilation; (2) ventilating the pit at the rates and for the time defined by the design aid simulations; (3) monitoring contaminant gas levels during pit ventilation until all contaminant gas levels are below the TLVs or PELs for hydrogen sulfide, carbon dioxide, methane, and ammonia, and oxygen levels reach 19.5% by volume; and (4) continuing to ventilate the pit and monitor contaminant and oxygen levels during the entire entry event.

The design aid simulation protocols are also useful to determine from which, if any, portions of barns above slotted-covered manure pits animals and personnel need to be evacuated prior to a pit ventilation event. The design aid is useful, also, for determining the maximum manure pit contamination gas levels below which such evacuation is not necessary. Such information is valuable because evacuation of animals often is a very time consuming and costly operation.

The current version of the online design tool does not include the influence of airflow obstructions, such as equipment, partitions, and animals, in the barn above a slotted-covered manure pit. Such obstructions would not influence the manure pit contaminant gas evacuation or oxygen replenishment times in the manure pit. However, the portions of the barn which need to be evacuated may be altered if these obstructions are extensive.

The design aid, including the input- and results-processing routines and the CFD software, is hosted on a Pennsylvania State University server. The online design aid is currently available to users at no cost. In the future, the design aid will be available to users either at no cost or for the cost of computer project simulation run time. This is extremely cost-effective, especially for the designer, planner, or regulatory personnel that only requires manure pit CFD simulations a few times each year.

## Author Contributions

HM: served as technical advisor to the project, especially for those aspects related to animal housing design, ventilation of animal housing systems, and manure pit layout. DH: developed protocols to collect and transform user project data into format compatible with SWFS; also developed protocols for extracting and reporting SWFS simulation outcomes; and also established the parameters under which SWFS simulations were conducted. DM: served as project director; provided oversight for all project activities; and provided technical guidance on the safety and regulatory issues related to the project. VP: provided technical guidance on project issues related to various aspects of numerical modeling, computer modeling, and computer simulation.

## Conflict of Interest Statement

The authors declare that the research was conducted in the absence of any commercial or financial relationships that could be construed as a potential conflict of interest.
